# The Effect of Different Ratios of Starch and Freeze–Thaw Treatment on the Properties of Konjac Glucomannan Gels

**DOI:** 10.3390/gels9020072

**Published:** 2023-01-17

**Authors:** Yangyang Wang, Jie Liu, Yawei Liu

**Affiliations:** College of Food Science and Engineering, Henan University of Technology, Zhengzhou 450001, China

**Keywords:** konjac glucomannan, starch, gelation, freeze–thaw, property

## Abstract

The composite gels of konjac glucomannan (KGM) and corn starch (CS) were prepared and treated by the freeze–thaw method. For KGM–CS gels, as the starch ratio rose from 0 to 100%, storage modulus (G′) decreased by 97.7% (from 3875.69 Pa to 87.72 Pa), degradation temperature decreased from 313.32 °C to 293.95 °C, and crystallinity decreased by 16.7%. For F–KGM–CS gels, G′ decreased by 99.0% (from 20,568.10 Pa to 204.09 Pa), degradation temperature increased from 289.68 °C to 298.07 °C, and crystallinity decreased by 17.1% with more starch content. The peak in infrared spectroscopy shifted to a higher wavenumber with more starch and to a lower wavenumber by freezing the corresponding composite gels. The detected retrogradation of the composite gels appeared for KGM–CS with 80% starch and F–KGM–CS with 40% starch. The endothermic enthalpy of free water rose by 10.6% and 10.1% with the increase in starch for KGM–CS and F–KGM–CS, respectively. The results of moisture distribution found that bound water migrated to free water and the water-binding capacity reduced with more starch. The results demonstrated that the molecular interaction in composite gels was weakened by starch and strengthened by freezing.

## 1. Introduction

Konjac glucomannan (KGM) is a natural polysaccharide extracted from the tuber of *Amorphophallus konjac C*. Koch. It has been widely used in food, chemical, pharmaceutical, cosmetic, coating, and painting industries [1,2]. KGM is composed of mannose and glucose with the molar ratio of 1.6:1 linked by glucosidic bonds β–(1–4), owning three branches every 32 sugar residues linked by glucosidic bonds β–(1–3) [3]. It is water-soluble due to about 1 in 19 units being acetylated in KGM [4,5]. In the presence of aqueous alkali with a pH range from 11.3 to 12.6, an irreversible phenomenon will happen, which is that KGM loses its acetyl groups [6]. The solubility of KGM exponentially declines as degree of substitution (DS) increases for high DS (0.5~3) KGM and improves with the increasing content of acetyl groups for comparatively low DS (0.05~0.42) KGM [5,6,7]. Compared with native KGM, deacetylation can facilitate the establishment of hydrogen bonds between KGM chains and show good gelation behavior of KGM [8,9]. Thus, KGM is usually used to prepare thermal irreversible gel by heating in a certain concentrated alkaline suspension with a temperature higher than 70 °C and does even not melt above 100 °C [10]. KGM is usually used with other polysaccharides, such as agarose [11], carrageenan, gum Arabic [12], and xanthan [13], to improve gel performance or form a compact network via a synergistic interaction.

Starch is one of the most important and abundant polysaccharides in nature and is usually used to prepare gel, due to its wide distribution, green production, and low cost. The starch gel is formed via the damage of starch granules due to the rearrangement of starch molecules by hydrogen bonds [14]. The property of starch gels can be enhanced by non-starch polysaccharides. The crystallinity and thermal degradation temperatures of starch–cellulose gel gradually increase with more cellulose content, indicating the decrease in amorphous regions [15,16]. The addition of carrageenan was beneficial to intermolecular crosslinking and an increasing elasticity of carrageenan–tapioca starch mixtures [17]. The stability of starch gels could be improved by the added 0.2% KGM [18]. KGM could effectively improve the viscoelasticity of corn starch due to its strong interaction in the composite system [2].

Freeze–thaw is an important technique to induce more interactions among biopolymer molecules and results in a denser and more aggregated network for hydrogels [19]. Iijima found that crosslinking points of galactomannan gel increased and molecular chains aggregated with freeze–thaw treatment [20]. A dense porous network microstructure emerged in the frozen KGM and starch-blended gels [21]. Freezing hardened the gels due to partial dehydration caused by the formation of ice crystals [22]. The freeze–thaw treatment increased the gel storage modulus of alginate gel almost 100 times [23] and provided higher mechanical strength for maltodextrin-agar gels [24].

This paper investigates the effect of different KGM/starch ratios and freeze–thaw treatments on the properties of the composite gels. The results could be used for optimal performance of the KGM–starch gels.

## 2. Results and Discussion

### 2.1. Rheological Analysis

#### 2.1.1. Frequency Sweep

**Figure 1:**
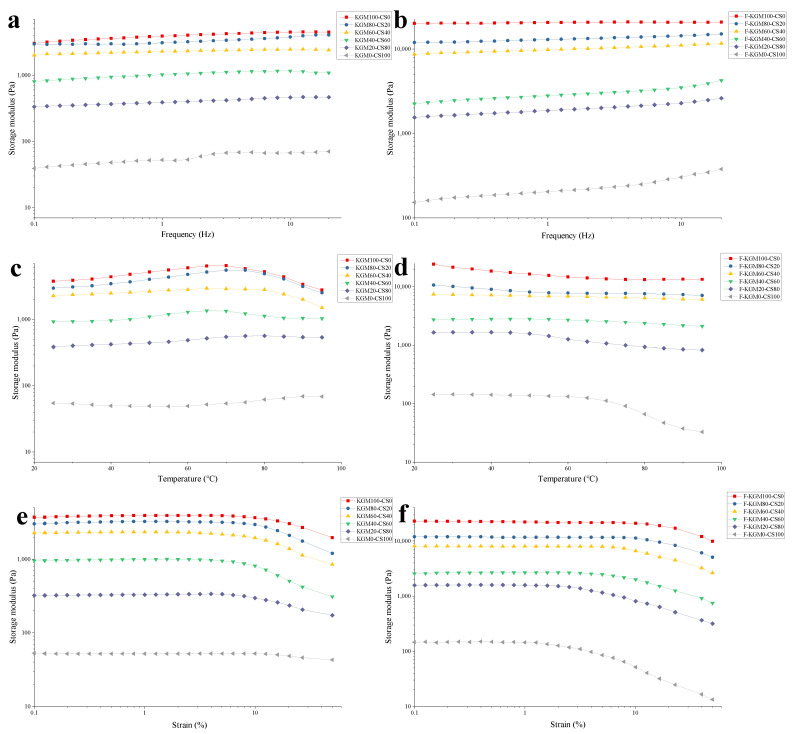
Rheological curves of the composite gels with different konjac glucomannan (KGM)/corn starch (CS) ratios. Frequency sweep of KGM–CS (**a**) and freeze–thawed KGM–CS (F–KGM–CS) (**b**) gels; temperature sweep of KGM–CS (**c**) and F–KGM–CS (**d**) gels; amplitude sweep of KGM–CS (**e**) and F–KGM–CS (**f**) gels.

The stable curve of G′ with increased frequency indicated that the frequency had no significant effect on KGM–CS and F–KGM–CS gels (Figure 1a,b) and the gels showed strong shearing resistance. As the KGM ratio rose from 0% to 100%, G′ of the composite gel increased from 87.72 Pa to 3875.69 Pa for KGM–CS and from 204.09 Pa to 20,568.10 Pa for F–KGM–CS, respectively. The transformation of weak gel to strong gel showed the synergistic effect of KGM with corn starch. Similar results were reported for gel-like CS–KGM and carrageenan/tapioca starch mixtures [2,17]. The G′ in frozen composite gels was higher than that in unfrozen gels, indicating that freezing treatment enhanced the rigidity of the gels. The effect of the freeze–thaw treatment might be that ice crystals resulted in more dense and aggregated polymer networks, in addition to the excellent elasticity of gels [19]. The results of the frequency sweep show that starch content had a negative effect and freeze–thaw treatment had a positive effect on the storage modulus.

#### 2.1.2. Temperature Sweep

The G′ increased when the gels occupied by KGM (KGM100–CS0 and KGM80–CS20) were heated to about 70 °C (Figure 1c), which conformed with the xanthan gum/tapioca starch mixture in which heating promoted the entanglement of polymer molecules [25]. The decrease in G′ with further heating was ascribed to that hydrogen bonds were weakened by rising temperature [26]. The G′ of KGM–CS gels was more stable with more starch during heating, indicating that KGM was more sensitive to heat than starch in the composite gels. The G′ of the gels declined with increasing starch content, suggesting that a more rigid structure was formed with more KGM in the mixtures.

The G′ of the gels was promoted by freeze–thaw treatment accordingly (Figure 1d). In the heating process, the disappearing rise in G′ of the freeze–thaw-treated gels occupied by KGM (KGM100–CS0, KGM80–CS20, and KGM60–CS40) implied no enhancement of entanglement for polymer molecules. The G′ of F–KGM–CS gels was more sensitive with more starch during heating, which was contrary to what happened to KGM–CS gels. This may be due to the destruction of the network arising from the freeze–thaw treatment being intensified by starch [25].

#### 2.1.3. Amplitude Sweep

At small strains, the stable storage modulus means that the inner structure of the composite gel was fully developed (Figure 1e,f). The three-dimensional network structure of the gel was able to resist the small strain amplitude; therefore, the moduli were independent of strain for different KGM–CS gels. In the linear viscoelastic region, the inner structure of the gel was reversibly deformed. When the applied strain increased, the storage modulus began to decrease slightly. The deformation of the structure was non-reversible, and the inner structure of the gels collapsed [27]. The starch-predominant gels resisted more strain values than the KGM-predominant gels. The yield strains of KGM–CS gels were as follows: 9.94% (KGM100–CS0), 7.92% (KGM80–CS20), 3.97% (KGM60–CS40), 5.02% (KGM40–CS60), 6.30% (KGM20–CS80), and 9.94% (KGM0–CS100) (Figure 1e). The yield strain decreased when starch ratio was lower than 40%, then it increased with a higher starch ratio.

The yield strains of F–KGM–CS gels (Figure 1f) decreased with rising starch ratio. The yield strains were as follows: 12.75% (F–KGM100–CS0), 10.05% (F–KGM80–CS20), 7.87% (F–KGM60–CS40), 5.01% (F–KGM40–CS60), 2.51% (F–KGM20–CS80), and 1.26% (F–KGM0–CS100). The storage moduli and the linear viscoelastic region were improved by the freeze–thaw treatment for the KGM-predominant gels. The longer linear viscoelastic region meant that the nodes in gels had a higher resistance to the applied deformation. Similar reports of KGM gels proved that the freeze–thaw treatment resulted in a significantly stiffer gel [28]. Though they had higher storage moduli compared to unfrozen gels, the shorter linear viscoelastic region for the starch-predominant gels indicated a somewhat distorted network structure, as water came out of the aggregated phase. Therefore, the gel properties were enhanced by the freeze–thaw treatment and weakened by starch.

### 2.2. TGA Analysis

**Figure 2:**
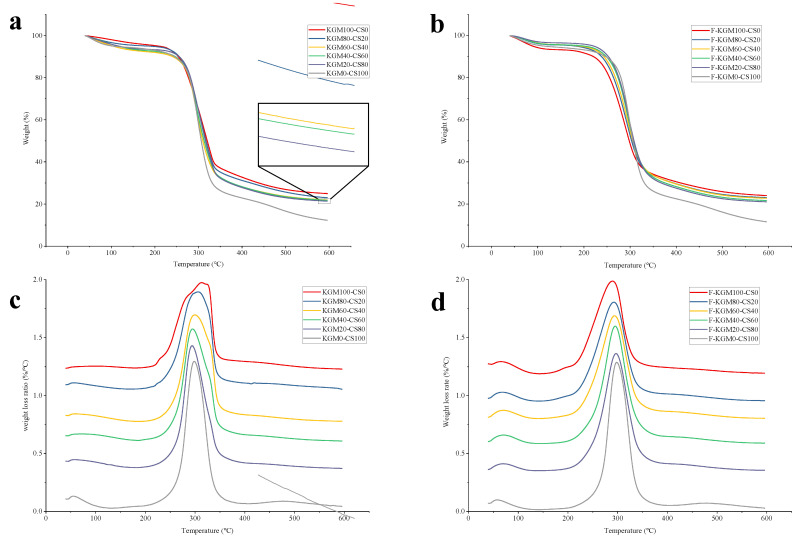
Thermal gravimetric graph of the composite gels with different KGM/starch ratios. The weight loss of KGM–CS (**a**) and F–KGM–CS (**b**) gels. The weight loss rate of KGM–CS (**c**) and F–KGM–CS (**d**) gels.

Thermal gravimetric analysis is widely used to determine the thermal stability of materials [29]. The weight loss around 300 °C (Figure 2a,b) was assigned to the dissociation of polymers, which was accordance with previous reports [30]. For KGM100–CS0, KGM80–CS20, KGM60–CS40, KGM40–CS60, KGM20–CS80, and KGM0–CS100, the weight loss ratios were 73.84 ± 0.26%, 75.76 ± 0.13%, 76.16 ± 0.21%, 76.43 ± 0.17%, 77.08 ± 0.14%, and 86.99 ± 0.34%, respectively. For the according freeze–thaw-treated gels, the weight loss ratios were 69.26 ± 0.32%, 72.45 ± 0.11%, 73.02 ± 0.08%, 73.98 ± 0.15%, 75.52 ± 0.06%, and 88.75 ± 0.29%, respectively. The weight loss ratio increased with more starch. It was demonstrated that the increased weight loss ratios of polymers were ascribed to the destruction of ordered crystals [16]. It could be inferred that CS did not interact synergistically with KGM to promote the formation of an ordered structure. The inference was similar to Yoshimura’s [31]. After freezing, numerous ice crystals extruded the aggregated polymer molecules and caused the dehydration of polymer molecules [19]. Thus, the ordered structure was strengthened, and the thermal stability was improved. The higher weight loss ratio of starch caused by the freeze–thaw treatment demonstrated the distorted structure suggested by the amplitude sweep results.

The degradation temperatures of KGM–CS gels were 313.32 ± 0.35 °C (KGM100–CS0), 305.77 ± 0.26 °C (KGM80–CS20), 299.45 ± 0.43 °C (KGM60–CS40), 294.88 ± 0.19 °C (KGM40–CS60), 293.95 ± 0.16 °C (KGM20–CS80), and 298.41 ± 0.39 °C (KGM0–CS100), respectively (Figure 2c). The reason for the decreased degradation temperature of KGM–CS gels was that starch in the composite gels weakened hydrogen bonds [32]. The degradation temperature of CS increased compared to KGM40–CS60 and KGM20–CS80, which revealed the crucial role of the ordered structure from starch [33]. The degradation temperatures of the according F–KGM–CS gels were 289.68 ± 0.07 °C, 292.39 ± 0.14 °C, 293.74 ± 0.22 °C, 295.05 ± 0.12 °C, 296.73 ± 0.21 °C, and 298.07 ± 0.24 °C, respectively (Figure 2d). The freeze–thaw treatment decreased the degradation temperature of the composite gels. As the starch ratio increased, the degradation temperature of F–KGM–CS gels rose, which was contrary to the declined degradation temperature of KGM–CS gels. KGM slowed down the retrogradation rate of starch [34]. The ice crystals were large in KGM and the composite gels, as there was more free water in their phases than that in retrograded starch. As a result, the network structure after thawing became quite distorted with large ice crystals.

For KGM–CS gels, more starch caused lower degradation temperatures and higher weight loss ratios, which suggested that starch was easy to dissociate compared to KGM. For F–KGM–CS gels, more starch caused higher both degradation temperatures and weight loss ratios, which suggested that the interaction between KGM and CS was promoted by the freeze–thaw treatment. Both the degradation temperature and weight loss ratio of F–KGM–CS gels were lower that of KGM–CS gels, which indicated that the composite gels became thermolabile due to some destruction of the network arising from the freeze–thaw treatment, while the interaction between the components was enhanced by freezing.

### 2.3. DSC Analysis

**Figure 3:**
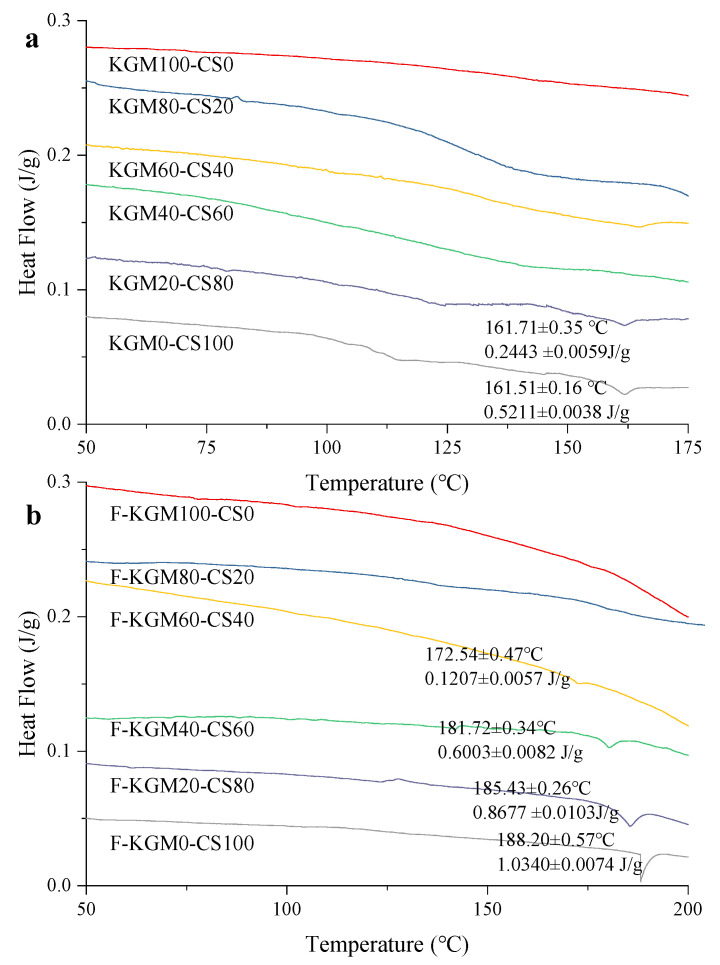
Retrogradation of starch in the composite gels: (**a**) KGM–CS gels; (**b**) F–KGM–CS gels.

Retrogradation is a common phenomenon in starch paste, which can be detected by DSC. Figure 3a,b show the retrogradation in KGM–CS and F–KGM–CS gels. The high gelatinization temperature was due to the retrogradation of amylose in the short term. The retrogradation could not be detected when the starch ratio was less than 80% in KGM–CS gels and 40% in F–KGM–CS gels. The results suggested that the more KGM there was in the composite gels, the more the depression effect on the starch retrogradation. The freeze–thaw treatment relieved the depression effect. Similar results were found in another study where sodium alginate could prevent the retrogradation of wheat starch [35].

The detected retrogradation was strengthened for KGM20–CS80 and KGM0–CS100 by the freezing, as both the peak temperatures and endothermic enthalpies of their frozen gels increased. In addition, in F–KGM–CS gels, the enhancement was promoted with more starch content. The results were consistent with the temperature sweep and TGA, in that freezing accelerated the interaction by physical crosslinking and hydrogen bonds in the junction zones of the polymeric network [36]. For both KGM–CS and F–KGM–CS gels, the syneresis of starch occurred easily with more starch and caused the obvious phase separation, which was in conformity with other reported starch–hydrocolloid combinations [37].

### 2.4. XRD Analysis

**Figure 4:**
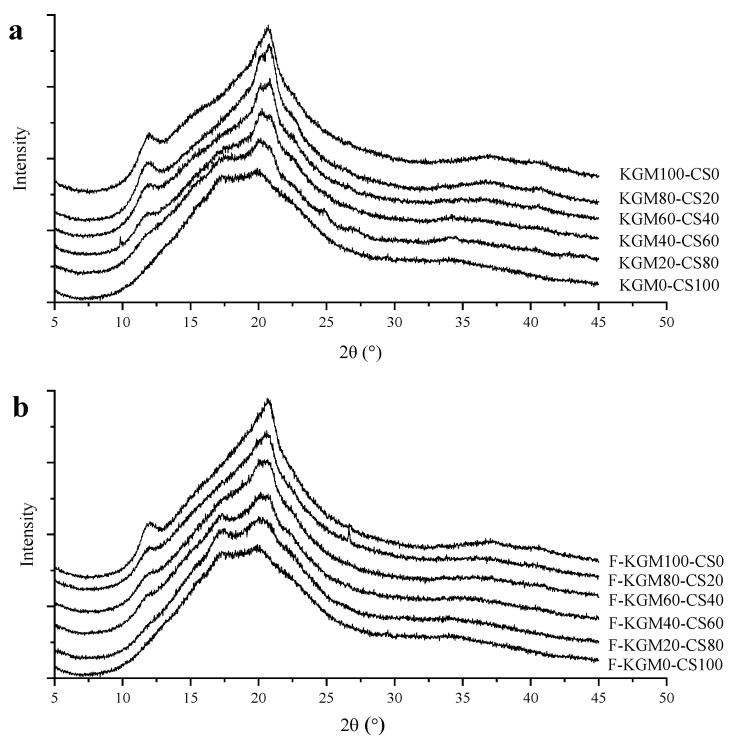
XRD profiles of the composite gels with different KGM/CS ratios: (**a**) KGM–CS gels; (**b**) F–KGM–CS gels.

The composite gels exhibited a broad diffuse scattering at about 20° (2θ), and there were distinctions in the position and strength of the diffuse scattering (Figure 4). There were two peaks at 12.03° and 20.71° shown in the profile of KGM (KGM100–CS0). A broad dispersion peak in native KGM at 2θ = 22° was reported [38], in addition to two weak peaks located around 2θ = 10.1° and 11.4° and other sharp diffraction peaks at 2θ = 19.4°, 20.5, and 21.8° [39]. The shifting of peaks and the different peak numbers might be due to the various sources and the effect of alkaline on its acetyl groups. KGM0–CS100 was the retrograded CS which owned two weak peaks at 17.27° and 20.00° in previous reports [40,41]. As starch ratio increased, the peak at 12.03° gradually diffused, the peak at 17.27° enhanced, and the peak at 20.71° shifted to 20.00°. Table 1 shows the crystallinity of KGM–CS and F–KGM–CS gels. The increase in starch ratio reduced the crystallinity of both the frozen and unfrozen composite gels. The results validated the above discussion of rheological and thermal properties and a previous report that KGM did not interact synergistically with starch to promote the formation of an ordered structure [31]. The crystallinity of F–KGM–CS gels was higher than that of unfrozen corresponding KGM–CS gels, which showed that freezing strengthened the ordered arrangement of composite gels. Therefore, KGM and freezing had positive effects on the crystallinity in composite gels.

### 2.5. FT–IR Analysis

**Figure 5:**
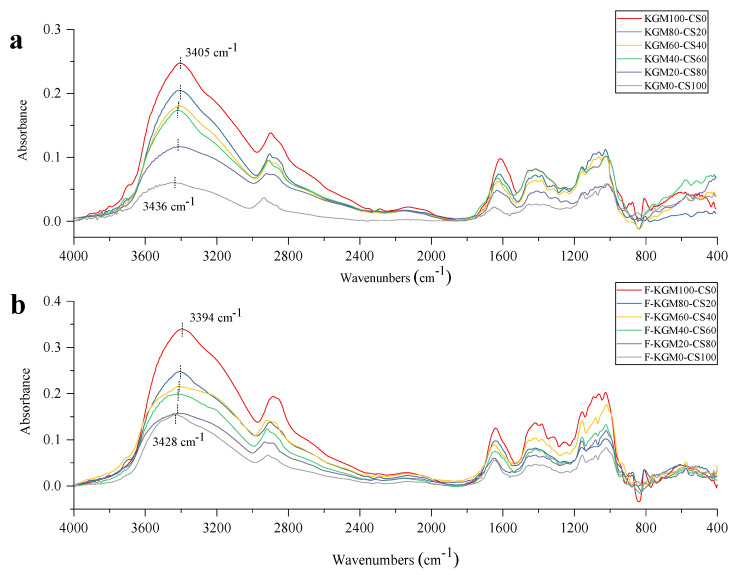
FT–IR spectra of the composite gels with different KGM/starch ratios: (**a**) KGM–CS gels; (**b**) F–KGM–CS gels.

There is a peak acetyl group at 1730 cm^−1^ in the spectrum of native KGM [9], which disappeared in the alkaline prepared composite gels (Figure 5). The removing of acetyl groups by adding NaOH and heating was beneficial to gelling. The peaks at 1025 cm^−1^ and 1062 cm^−1^ represented the stretching vibration of C–O–C groups. The peaks at 1383 cm^−1^ and 2923 cm^−1^ were from the –CH2– groups [30,42,43]. The absorption peaks at 808 cm^−1^ and 874 cm^−1^ assigned to the mannose of KGM weakened and disappeared when the starch ratio reached 100%. The absorption peaks at 761 cm^−1^, 850 cm^−1^, and 932 cm^−1^ assigned to the α–configuration of glycosidic linkage in starch were strengthened with more starch in the composite gels.

The main difference in FT–IR was the stretching vibration of –OH. As starch ratio increased, the peak corresponding to the stretching vibration of –OH migrated from 3405 cm^−1^ to 3436 cm^−1^ in KGM–CS gels (Figure 5a), and from 3394 cm^−1^ to 3428 cm^−1^ in F–KGM–CS gels (Figure 5b). The peak shifted to a higher wave number with an increased starch ratio, indicating the gradual weakness of hydrogen bonds [44]. As the strength of KGM and the starch mixture was maintained by hydrogen bonds [45], the weakened hydrogen bonds could explain the reason for the decreased G′, crystallinity, and thermal stability with increased starch ratio. The peak shifted to a lower wavenumber by freezing the corresponding composite gels, suggesting a stronger hydrogen bond [28]. This supports the rheological, thermal, and crystallinity results.

### 2.6. Moisture Distribution in the Composite Gels

**Figure 6:**
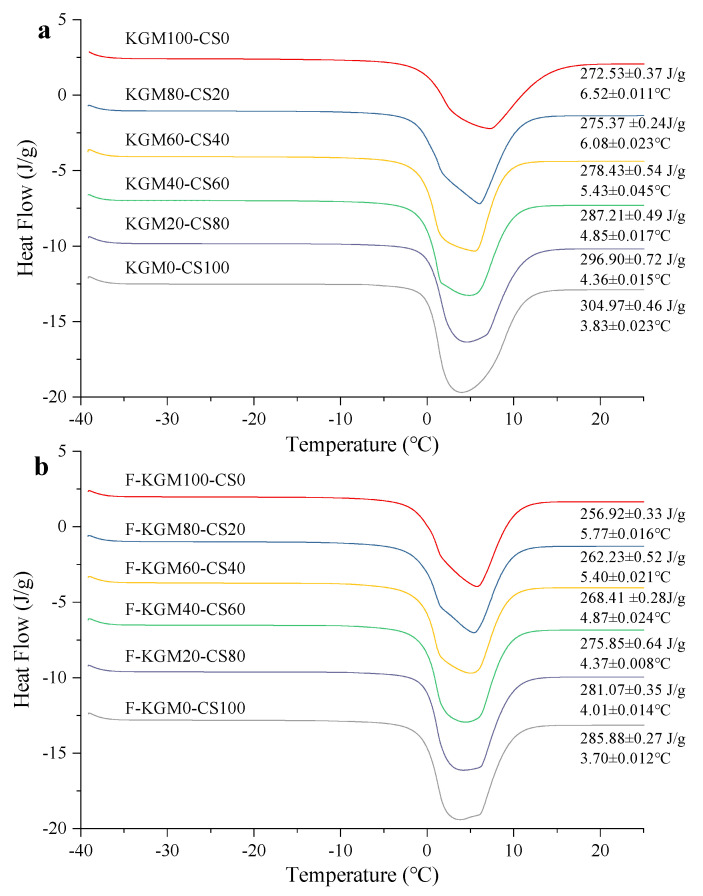
The melting of free water in the composite gels with different KGM/starch ratios: (**a**) KGM–CS gels; (**b**) F–KGM–CS gels.

The state of water can provide useful information on the effect of KGM/CS ratio and freezing on the behavior of composite gels. According to the thermodynamic properties, water molecules absorbed by the hydrophilic region are known to be present in three conditions: (1) free water with a melting temperature at 0 °C, (2) freezable bound water with a melting temperature at lower than 0 °C, and (3) non-freezable bound water, which did not crystallize even at −40 °C [20]. The existence of endothermic peaks in KGM–CS and F–KGM–CS gels was considered as free water because their melting temperatures were around 0 °C, and no endothermic peaks of freezable bound water were present (Figure 6). For both KGM–CS gels (Figure 6a) and F–KGM–CS gels (Figure 6b), as the starch ratio increased, the endothermic enthalpy increased and the melting temperature reduced. As the differences in quantity of freezable and non-freezable water probably originated due to the order–disorder structure, the crystalline structure played an important role in inhibiting the penetration of water into the amorphous region [46]. The declining crystalline content in the composite gels caused by more starch led to the penetration of water, so the excess water was easily crystallized and eventually melted during heating. In addition, both the endothermic enthalpy and melting temperatures of F–KGM–CS gels were lower than that of KGM–CS gels. It was reasonable that the increased crystalline contents in the frozen gels caused a low quantity of freezable water, and the strengthened interaction by freezing prevented the movement of water.

**Figure 7:**
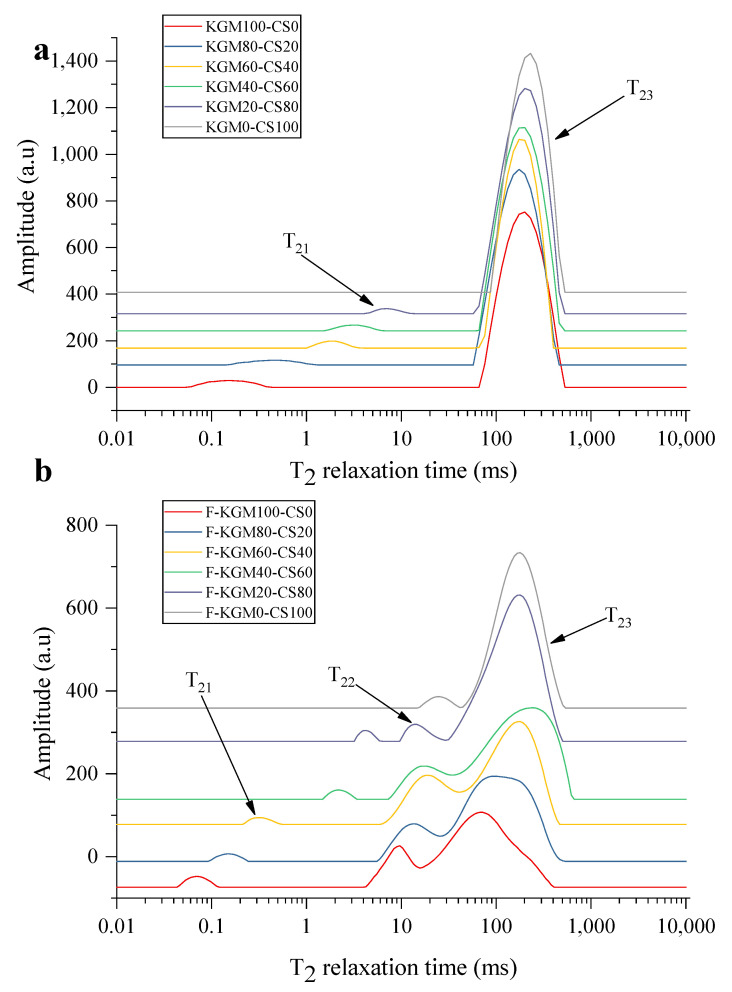
The distribution of the water relaxation times (T_2_) of the composite gels with different KGM/starch ratios: (**a**) KGM–CS gels; (**b**) F–KGM–CS gels.

Moreover, the LF–NMR analysis was further carried out to study the moisture distribution. According to the weakly bound water (1 ms < T_2_ < 100 ms), including T_21_ (the peak time of relaxation time below 10 ms), T_22_ (the peak time of relaxation time between 10 ms and 100 ms), and T_23_ (the peak time around 100 ms) [4,47,48], the water in KGM–CS gels could be divided into bound water (T_21_) and free water (T_23_) (Figure 7a). The prolonged relaxation time of T_21_ with the risen starch ratio meant that the combining capacity between water molecules and polymers in KGM–CS gels weakened. The enlarged peak area of T_23_ indicated that the free water content in KGM–CS gels increased with added starch. This suggests that starch destroyed the interaction between water and polymers, then caused the migration of bound water and more free water. For F–KGM–CS gels, water could be divided into tightly bound water (T_21_), weakly bound water (T_22_) and free water (T_23_) (Figure 7b). The T_21_ peak showed longer relaxation times with more starch, indicating that the binding capacity between water and polymers weakened. The peak area of T_23_ was enlarged with increased starch ratio, indicating that the free water content rose. Compared with KGM–CS gels, a significant difference was that a new peak (T_22_) appeared in the F–KGM–CS gels. As the starch ratio increased, the peak area of T_22_ reduced and its relaxation time rose. The implication that bound water migrated to free water by starch through the weakly bound water transition was consistent with unfrozen gels.

The results of both DSC and LF–NMR analysis concluded that the free water in gels rose and the water-binding capacity reduced as starch ratio increased. More free water weakened the formation of strong intermolecular hydrogen bonds [49]. This confirmed that the interaction of composite gels was weakened by starch and strengthened by freezing.

## 3. Conclusions

The rheological and thermal properties, crystallinity, and moisture distribution of the composite gels with or without the freeze–thaw treatment were analyzed to explore the effect of the ratio of KGM and CS and freezing on gelling behavior. The results demonstrated that the molecular interaction of composite gels was inhibited by starch and strengthened by freezing. This study will be a driving force to understand the influences of KGM, starch, and freezing on the composite gels, which are beneficial to develop gel functions and reduce costs, such as in low-calorie foods, meat alternatives, and the encapsulation and delivery of aroma compounds.

## 4. Materials and Methods

### 4.1. Materials

Native KGM flour (KJ30, 77.24% of purity) was purchased from Konson Konjac Science and Technology Co., Ltd. (Ezhou, China), and corn starch (CS) (31.95% of amylose content) was from Huifeng biotechnology Co., Ltd. (Zhumadian, China). 3,5–DNS, formic acid, glucose, phenolsodium hydroxide, sodium hydrogen sulfite, seignette salt, and sulfuric acid were purchased from Kermel Chemical Reagent Co., Ltd. (Tianjin, China).

### 4.2. Preparation of KGM–CS Gels and Freeze–Thaw-Treated Gels (F–KGM–CS)

Starch was precisely weighed and dispersed in alkali solution (adjusted by NaOH, pH = 12.3) at 25 °C by magnetic stirring (150 rpm). Starch slurry was heated (95 °C) in water bath for 90 s and then cooled to 25 °C. KGM flour was added slowly to starch solution. The mixture was kept stirring and heated at 80 °C for 80 min, followed by standing at 25 °C for 24 h to stabilize the KGM–CS gels. A series of composite gels were obtained by changing KGM/CS ratio: 100/0, 80/20, 60/40, 40/60, 20/80, and 0/100 (marked as KGM100–CS0, KGM80–CS20, KGM60–CS40, KGM40–CS60, KGM20–CS80, and KGM0–CS100). The composite gels were frozen (−18 °C) for 24 h, then thawed at 25 °C for 2 h to obtain the F–KGM–CS gels. Accordingly, they were marked as F–KGM100–CS0, F–KGM80–CS20, F–KGM60–CS40, F–KGM40–CS60, F–KGM20–CS80, and F–KGM0–CS100.

### 4.3. Rheological Measurements

The rheological measurement was performed using controlled strain rheometer (DISCOVERY HR–1, TA Instruments, New Castle, DE, USA) with a parallel plate (25 mm diameter, 1 mm gap). Temperature was controlled by peltier steel plate. The samples were covered with silicone oil to prevent water evaporation. The linear viscoelastic region of composite gels was determined by the amplitude sweep measurement from 0.1% to 50% with constant frequency (1 Hz) at 25 °C. After amplitude sweep, 1% strain was chosen for further examination. The frequency sweep was obtained from 0.1 Hz to 20 Hz and temperature sweep was conducted from 25 °C to 95 °C with a heating rate of 5 °C/min.

### 4.4. Thermal Gravimetric Analysis (TGA)

TGA measurement of composite gels was carried out by TA Q50 apparatus (TA Instruments, New Castle, DE, USA). Lyophilized sample (10 mg) was weighed and deposited in the platinum basket. The sample was heated from 40 °C to 600 °C with a heating rate of 10 °C/min under nitrogen atmosphere at a constant flow rate of 40 mL/min.

### 4.5. Differential Scanning Calorimetry (DSC)

DSC measurement was carried out with TA Analysis software (Trios 4.4.0, TA Instruments, New Castle, USA). The system was calibrated with pure indium. TA Tzero aluminum pans were used, and an empty pan was the reference. The samples (10 mg) were cooled to −40 °C and then heated to 40 °C at a rate of 5 °C/min with the gas (N2) at 40 mL/min to measure the free water content in composite gels. In addition, the samples were heated from 40 °C to 200 °C to measure retrogradation of composite gels.

### 4.6. X-ray Diffraction (XRD)

The crystallinity of the powder of the composite gels was carried out by D8 DVANCE (Bruker Corporation, Germany). Samples were analyzed from 2θ of 5° to 45° with a scanning rate of 4°/min. The experiment condition was 0.02° of step size, 40 kV of voltage, and 30 mA of current.

### 4.7. Fourier Transform Infrared Spectroscopy (FT–IR)

Composite gels were lyophilized and ground to powder. FT–IR analysis was carried out from 400 cm^−1^ to 4000 cm^−1^ by FT–IR spectrometer (Nicolet 6700, Thermo Scientific, Waltham, MA, USA). The dried KBr and 1% samples were mixed to prepare discs for FT–IR analysis.

### 4.8. Low-Field Nuclear Magnetic Resonance (LF–NMR)

Moisture distributions of the composite gels were measured by LF–NMR analyzer (MicroMR–CL–I, Niumag Analytical Instrument Co., Ltd., Suzhou, China) under Carr–Purcell–Meiboom–Gill (CPMG) sequences. Approximately 0.5 g of composite gel was transferred to a 10 mm diameter NMR glass tube. The measured parameters of transverse relaxation time (T_2_) were as follows: 4000 ms of waiting time, 0.2 ms of echo time, 18,000 echo numbers, and 16 scan numbers.

### 4.9. Statistical Analysis

The experiments were carried out in triplicate and the results were presented by means and standard deviations. Analysis of variance (ANOVA) was processed using SPSS 24.0 (SPSS Inc., Chicago, IL, USA).

## Figures and Tables

**Table 1:** The crystallinity of composite gels.

KGM/CS Ratio	Crystallinity of KGM–CS Gels (%)	Crystallinity of F–KGM–CS Gels (%)
100/0	19.8 ± 0.07	20.5 ± 0.03
80/20	19.5 ± 0.02	19.9 ± 0.05
60/40	19.3 ± 0.03	19.4 ± 0.09
40/60	18.6 ± 0.06	18.9 ± 0.07
20/80	17.9 ± 0.09	18.3 ± 0.13
0/100	16.5 ± 0.12	17.0 ± 0.21

## Data Availability

Not applicable.
